# Breakthrough infections and incomplete vaccine efficacy drive pathogen immune escape

**DOI:** 10.1371/journal.pone.0356544

**Published:** 2026-09-02

**Authors:** Minjin Kim, Eunha Shim

**Affiliations:** Department of Mathematics, Soongsil University, Dongjak-gu, Seoul, Republic of Korea; The University of New Mexico, UNITED STATES OF AMERICA

## Abstract

**Introduction:**

While vaccines effectively reduce disease severity and mortality, their consequences for pathogen immune escape remain poorly understood. Vaccines provide protection through three distinct mechanisms: prevention of infection, reduction of symptoms, and limitation of transmission. When vaccine efficacy is incomplete across multiple dimensions, breakthrough infections in vaccinated individuals may paradoxically increase pathogen escape pressure. This study investigates how vaccine efficacy profiles and coverage levels jointly affect escape pressure.

**Materials and methods:**

We developed a deterministic compartmental transmission model incorporating three distinct vaccine efficacy parameters: prevention of infection, reduction of symptoms, and limitation of transmission. The model quantifies differential contributions of symptomatic and asymptomatic infections in vaccinated and unvaccinated populations to immune escape pressure.

**Results:**

Infection-blocking, symptom-blocking, and transmission-blocking immunity each reduced escape pressure, although no single component alone was sufficient to suppress escape pressure across most conditions. Combinations of efficacy components produced greater reductions. When vaccine efficacy was low across multiple components, increasing coverage could paradoxically elevate escape pressure above pre-vaccination levels. Even in a fully vaccinated population, escape pressure could exceed the pre-vaccination level when infection-blocking and symptom-blocking efficacy were simultaneously low. The effectiveness of symptom-blocking immunity depended on infection-blocking efficacy — when infection-blocking efficacy is low, symptom-blocking efficacy alone did not always suppress escape pressure below pre-vaccination levels.

**Discussion:**

The effect of vaccination on immune escape depends on both vaccine efficacy profiles and coverage levels. Increasing vaccination coverage may increase rather than reduce escape pressure when efficacy is low across multiple dimensions. Combining efficacy across multiple dimensions produced greater reductions in escape pressure than relying on a single efficacy component.

## Introduction

During the course of infectious disease spread, pathogen variants continually evolve, with selective pressures often favoring traits that enhance transmission rates, extend pre-symptomatic periods, and sometimes lower disease severity [1]. However, mutations affecting transmissibility, disease severity, and immune evasion can interact in complex ways, making evolutionary trajectories difficult to predict [2]. The continual emergence of variants with increased transmissibility and immune escape can reduce the effectiveness of public health measures and vaccination campaigns [3].

Pertussis provides an example of pathogen adaptation under vaccine-induced selection pressure. Pertussis is a highly infectious respiratory disease caused by *Bordetella pertussis* [4]. The introduction of whole-cell pertussis vaccines substantially reduced global incidence, but concerns over the high reactogenicity of these vaccines led to the development of acellular vaccines as a safer alternative. Recently, several countries with high coverage of acellular vaccines have reported a resurgence of pertussis [4]. While this resurgence likely depends on several factors — including improved laboratory diagnostics, enhanced surveillance, and waning immunity following vaccination [4] — the epidemiological pattern of resurgence coinciding with acellular vaccine introduction suggests that these vaccines favor adaptation of *Bordetella pertussis* toward vaccine escape [3–5].

Genetic variation arises from replication errors during bacterial reproduction. Selective pressures from host immune responses and vaccine-induced immunity then act on this variation, favoring variants with greater immune-evasion capabilities [6]. Variant emergence is particularly prominent in populations where herd immunity is not fully achieved [6]. Empirical studies have demonstrated that vaccines can exert selective pressure favoring new variants. In Australia, acellular pertussis vaccines were introduced in 1997 [7]. During the 2008–2012 pertussis outbreak, some clinical isolates were found not to express pertactin, a component of acellular vaccines [7]. Most of these isolates carried alleles representative of the strains circulating in Australia, suggesting that *Bordetella pertussis* has been continually evolving in response to vaccine-induced escape pressure [7].

A theoretical study by Gutierrez and Gog explored the role of vaccination in pathogen evolution [8], emphasizing the relative contribution of infections in vaccinated versus unvaccinated populations to variant emergence. Mathematical modeling has demonstrated that vaccination strategies may either reduce or paradoxically increase escape pressure, depending on vaccine efficacy profiles and coverage levels [9,10]. However, it remains unclear how escape pressure is shaped by the interaction between breakthrough infections (i.e., infections despite vaccination) and the three dimensions of vaccine efficacy (infection-blocking, symptom-blocking, and transmission-blocking).

Asymptomatic infections account for up to 56% of pertussis infections, but their contribution to escape dynamics remains unclear [11,12]. For example, asymptomatic infections were not considered in the model of Gutierrez and Gog [8], and models that do include them [13,14] have not examined how varying contributions from asymptomatic infections to escape pressure interact with the three-way decomposition of vaccine efficacy under different coverage levels [15].

We developed a compartmental transmission model incorporating three independent vaccine efficacy parameters — prevention of infection, reduction of symptoms, and limitation of transmission — and used pertussis-specific parameters to examine how these three efficacy dimensions and vaccination coverage affect escape pressure [8,16]. We also identified conditions under which increasing coverage may increase escape pressure.

## Materials and methods

### Epidemic models

**Fig 1:**
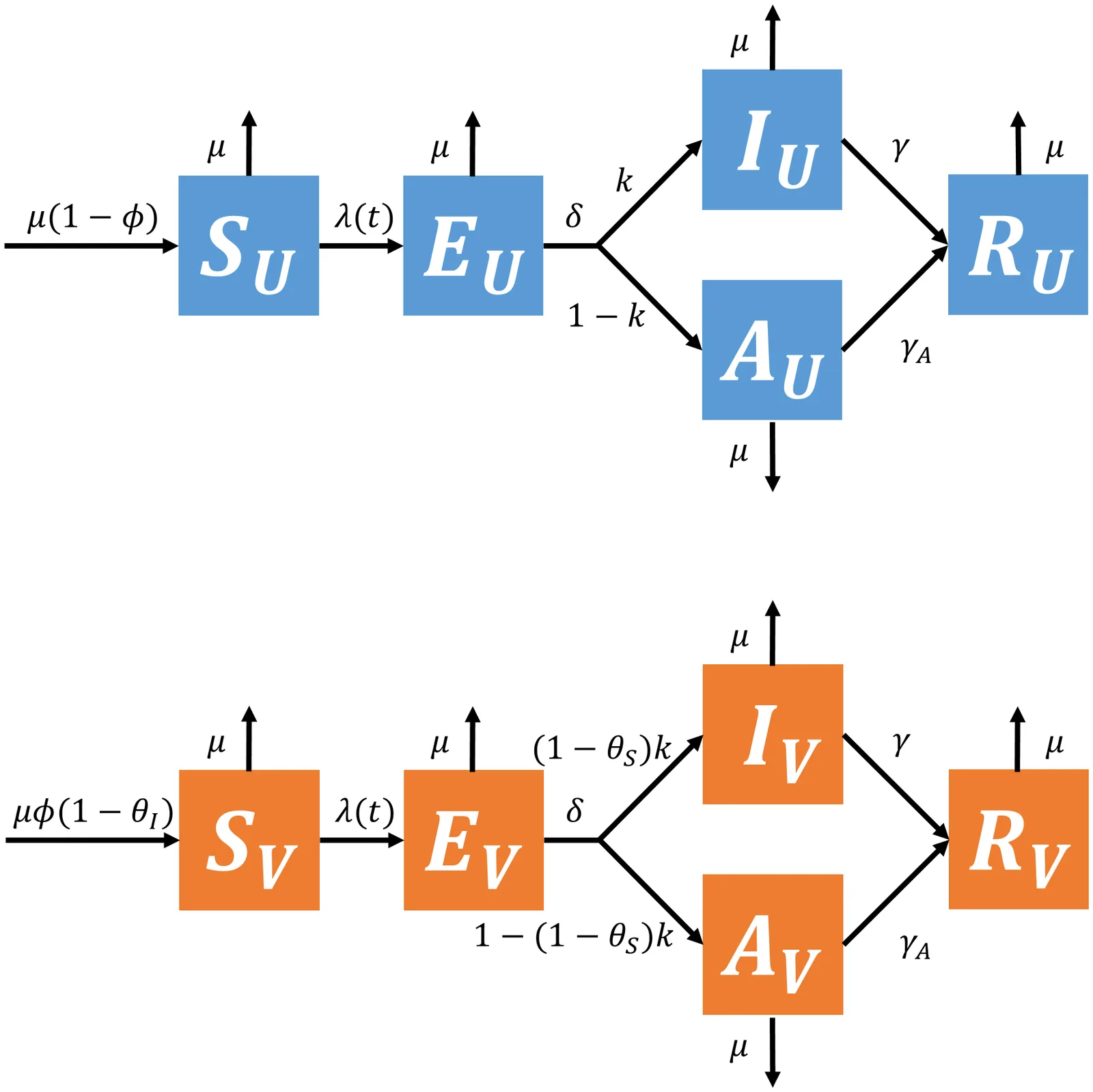
Diagram of a transmission dynamic model with vaccination and asymptomatic infection. Orange compartments represent vaccinated groups, while blue compartments represent unvaccinated groups. The proportion of the population, $\mu \phi \theta_{I}$, who are fully immunized and gain complete immunity, is not shown in the diagram.

We modeled the spread of infection in a vaccinated population, incorporating both symptomatic and asymptomatic transmission. The population was assumed to be well mixed and divided into 11 compartments based on disease and vaccination status. To maintain a stable population size, birth and death rates were assumed to be identical, denoted as μ per capita (Fig 1). The parameter μ was set to $\text{3}\times {\text{1}\text{0}}^{-\text{5}}$ (day^-1^) based on an average human life expectancy [17].

We assumed three types of vaccine effects: protection against infection, reduction in symptom development, and limitation in transmission probability. A proportion (ϕ) of newborns is vaccinated at birth. A proportion $\theta_{I}$ of this vaccinated population was assumed to acquire full lifelong immunity, whereas the remainder remain susceptible to infection, but still benefit from the vaccine through reduced probability of developing symptoms ($\theta_{S}$) in breakthrough infections and reduced transmission probability ($\theta_{T}$). The three efficacy parameters ($\theta_{I}$, $\theta_{S}$ and $\theta_{T}$) range from 0 (no protection) to 1 (complete protection). Vaccination is not assumed to shorten the duration of breakthrough infections.

The unvaccinated population is subdivided into the following compartments: susceptible ($S_{U}$), exposed ($E_{U}$), asymptomatic and infectious ($A_{U}$), symptomatic and infectious ($I_{U}$), and recovered compartments ($R_{U}$). Similarly, the proportion of the vaccinated population that has not obtained full immunity is subdivided into susceptible ($S_{V}$), exposed ($E_{V}$), asymptomatic and infectious ($A_{V}$), symptomatic and infectious ($I_{V}$), and recovered compartments ($R_{V}$). Birth rates into the $S_{U}$ and $S_{V}$ compartments are μ(1−ϕ) and $\mu \phi (\text{1}-\theta_{I})$, respectively. Finally, the vaccinated population with full immunity is denoted by V.

The exposed compartment represents the latent period following infection, during which transmission does not occur. After an average of $\delta^{-\text{1}}$ days, a proportion k of exposed individuals progresses to symptomatic infection (I), while the remaining proportion (1−k) progresses to asymptomatic infection (A). Recovery from symptomatic and asymptomatic infection is assumed to occur after an average of $\gamma^{-\text{1}}$ days and $\gamma_{A}^{-\text{1}}\;$days, respectively, independent of vaccination status. Based on experimental observations for pertussis, we assumed that the mean time in the exposed compartment ($\delta^{-\text{1}}$) was 8 days and the average infection period was 63 days for symptomatic cases ($\gamma^{-\text{1}}$) and 21 days for asymptomatic cases ($\gamma_{A}^{-\text{1}}$) [18,19].

Symptomatic and asymptomatic infections are assumed to have different transmission rates, represented by β and $\rho_{A}\beta$, respectively, where $\rho_{A}$ denotes the relative infectiousness of asymptomatic infections compared to symptomatic ones. Asymptomatic carriers may maintain higher transmission rates since they do not experience symptoms that restrict social interactions. However, asymptomatic infections also exhibit significantly lower pathogen loads compared to symptomatic infections, which directly constrains both transmission potential and mutation generation capacity [20]. These antagonistic effects suggest that equal transmissibility between asymptomatic and symptomatic infections is a plausible baseline assumption, which is also supported by experimental and modeling studies [21–24], although empirical data on $\rho_{A}$ for pertussis remain limited. We assumed equal transmissibility ($\rho_{A}=\text{1}$) as our baseline and explored a range of $\rho_{A}$ values in sensitivity analyses (Fig 5). Model parameters and baseline values are summarized in Table 1.

**Table 1:** Description of model parameters.

Parameter	Description	Baseline value	References
$R_{\text{0}}$	Basic reproduction number	5.5	[25]
μ	Birth and death rate (day^-1^)	${\text{3}.\text{0}\times \text{1}\text{0}}^{-\text{5}}$	[17]
β	Transmission rate	${\text{8}.\text{7}\times \text{1}\text{0}}^{-\text{2}}$	Fitted to $R_{\text{0}}=\text{5}.\text{5}$
$\delta^{-\text{1}}$	Average duration of the latent period (days)	8	[18]
$\gamma^{-\text{1}}$	Average duration of a symptomatic infection (days)	63	[19]
$\gamma_{A}^{-\text{1}}$	Average duration of an asymptomatic infection (days)	21	[19]
k	Proportion of exposed individuals who become symptomatic	0.56	[11,12]
$\rho_{A}$	Relative infectiousness of asymptomatic infections compared to symptomatic infections	1.0	[23,26]
$\chi_{A}$	Relative contribution of asymptomatic infections to escape pressure compared to symptomatic infections	1.0	Assumed
ξ	Relative contribution of vaccinated infections to escape pressure compared to unvaccinated infections	2.0	Assumed
$\theta_{I}$	Vaccine efficacy in preventing infection	0.4	Assumed
$\theta_{S}$	Vaccine efficacy in preventing symptomatic infection	0.85	[27]
$\theta_{T}$	Vaccine efficacy in reducing transmission of breakthrough infections	0.1	Assumed
ϕ	Vaccination coverage level	0–1	Assumed

**Fig 2:**
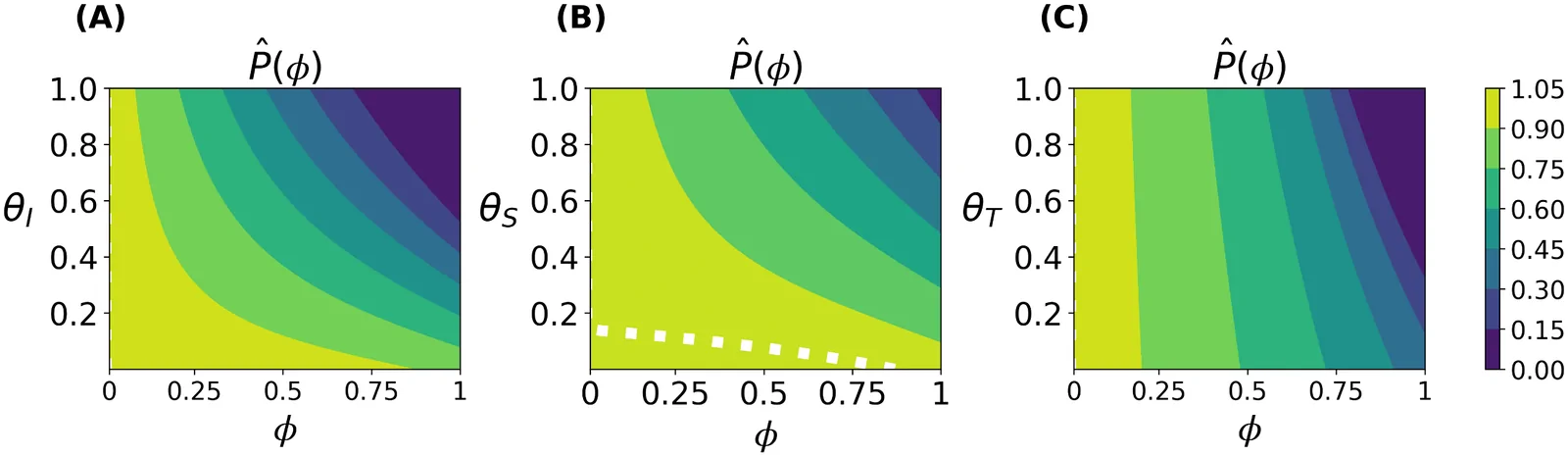
Impact of vaccine efficacies and coverage level on relative escape pressure. (A) Relative escape pressure $\hat{P}(\phi )$ with varying vaccination coverage level (ϕ) and vaccine efficacy to prevent infection ($\theta_{I}$). (B) Relative escape pressure $\hat{P}(\phi )$ with varying vaccination coverage level (ϕ) and vaccine efficacy to prevent symptomatic infection ($\theta_{S}$). (C) Relative escape pressure $\hat{P}(\phi )$ with varying vaccination coverage level (ϕ) and vaccine efficacy to reduce transmission of breakthrough infections ($\theta_{T}$). The white dotted curve in (B) represents $\hat{P}(\phi )=\text{1}$, indicating that the escape pressure at vaccination coverage ϕ is equal to the pre-vaccination level. Other parameters are set to the baseline values from Table 1: $R_{\text{0}}=\text{5}.\text{5},\;\mu ={\text{3}.\text{0}\times \text{1}\text{0}}^{-\text{5}},\delta^{-\text{1}}=\text{8},\;\gamma^{-\text{1}}=\text{6}\text{3},\;\gamma_{A}^{-\text{1}}=\text{2}\text{1},\;k=\text{0}.\text{5}\text{6},\;\rho_{A}=\text{1},\;\chi_{A}=\text{1},\;\xi =\text{2},\;\theta_{I}=\text{0}.\text{4},\;\theta_{S}=\text{0}.\text{8}\text{5},\;\theta_{T}=\text{0}.\text{1}$.

**Fig 3:**
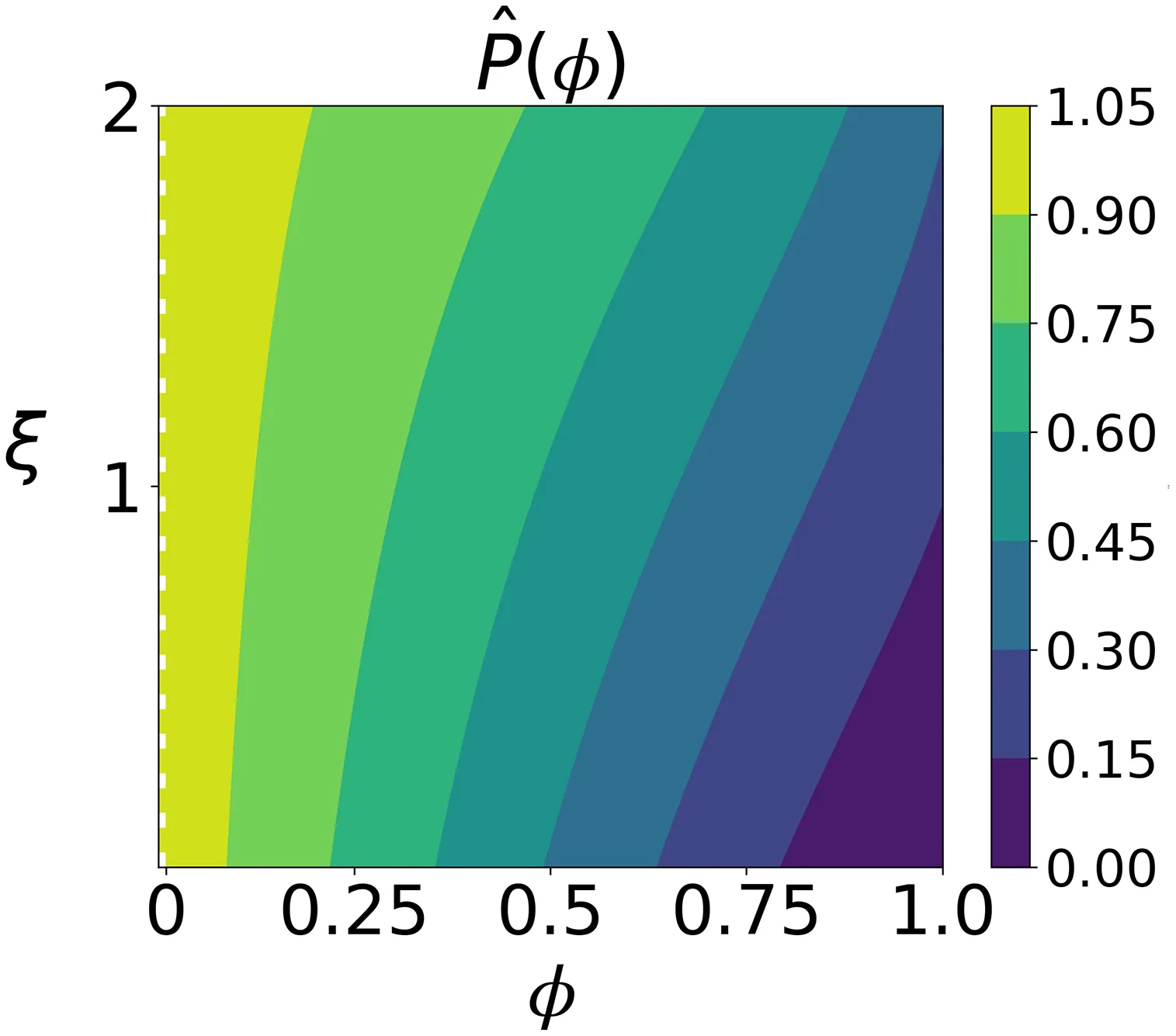
Relative escape pressure $\hat{P}\left(\phi \right)$ with varying relative contribution of breakthrough infections to the escape pressure (ξ) and vaccination coverage level (ϕ). The white dotted curve represents $\hat{P}(\phi )=\text{1}$, indicating that the escape pressure at vaccination coverage ϕ is the same as without vaccination. Other parameters are set to the baseline values from Table 1.

**Fig 4:**
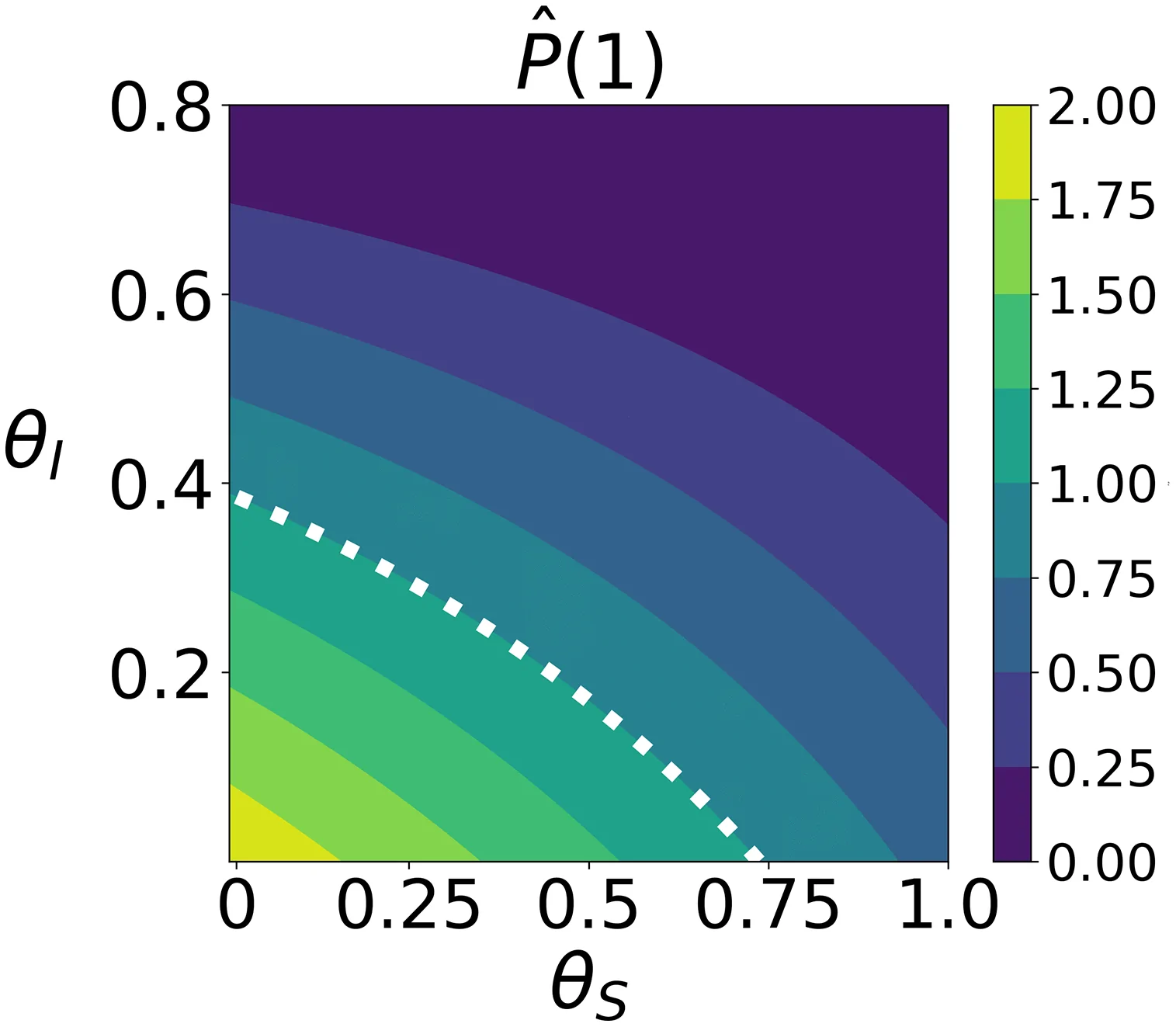
Relative escape pressure $\hat{P}\left(1\right)$ with varying vaccine efficacy against symptoms ($\theta_{S}$) and infection ($\theta_{I}$) in a fully vaccinated population. The white dotted curve represents $\hat{P}(\text{1})=\text{1}$, indicating that the escape pressure at full vaccination is the same as without vaccination. Other parameters are set to the baseline values from Table 1.

The system of ordinary differential equations governing the dynamics of the susceptible, exposed, infectious, and recovered compartments in both vaccinated and unvaccinated populations is described below. The disease transmission model for the unvaccinated population is:


$$\begin{array}{c} {\dot{S}}_{U}=\mu (\text{1}-\phi )-\left(\lambda (t)+\mu \right)S_{U} \end{array}$$

$$\begin{array}{c} {\dot{E}}_{U}=\lambda (t)S_{U}-(\delta +\mu )E_{U} \end{array}$$

$$\begin{array}{c} {\dot{A}}_{U}=\delta (\text{1}-k)E_{U}-\left(\gamma_{A}+\mu \right)A_{U} \end{array}$$

$$\begin{array}{c} {\dot{I}}_{U}=\delta kE_{U}-(\gamma +\mu )I_{U} \end{array}$$

$$\begin{array}{c} {\dot{R}}_{U}=\gamma_{A}A_{U}+\gamma I_{U}-\mu R_{U} \end{array}$$ (1)

The dynamics for the vaccinated compartments are:


$$\begin{array}{c} {\dot{S}}_{V}=\mu \phi (\text{1}-\theta_{I})-\left(\lambda (t)+\mu \right)S_{V} \end{array}$$

$$\begin{array}{c} {\dot{E}}_{V}=\lambda (t)S_{V}-(\delta +\mu )E_{V} \end{array}$$

$$\begin{array}{c} {\dot{A}}_{V}=\delta \left\{\text{1}-\left(\text{1}-\theta_{S}\right)k\right\}E_{V}-\left(\gamma_{A}+\mu \right)A_{V} \end{array}$$

$$\begin{array}{c} {\dot{I}}_{V}=\delta \left(\text{1}-\theta_{S}\right)kE_{V}-(\gamma +\mu )I_{V} \end{array}$$

$$\begin{array}{c} {\dot{R}}_{V}=\gamma_{A}A_{V}+\gamma I_{V}-\mu R_{V} \end{array}$$ (2)

The force of infection, λ(t), was given by


$$\begin{array}{c} \lambda (t)=\beta \{\rho_{A}A_{U}+I_{U}+(\text{1}-\theta_{T})(\rho_{A}A_{V}+I_{V})\}\text{.} \end{array}$$ (3)

Initial conditions were set to $S_{U}(\text{0})=\text{1}-\phi$, $S_{V}(\text{0})=\phi (\text{1}-\theta_{I})$, $R_{U}(\text{0})=R_{V}(\text{0})=\text{0}$, with a small number of infections. The fully protected subpopulation, denoted as V, follows the equation


$$\begin{array}{c} \dot{V}=\mu \phi \theta_{I}-\mu V \end{array}$$

with initial condition $V(\text{0})=\phi \theta_{I}$, leading to the steady state, $V^{*}=\phi \theta_{I}$. This fully protected population $\mu \phi \theta_{I}$ does not interact with any of the other compartments. The basic reproduction number, $R_{\text{0}}$, is defined as the average number of secondary cases generated by a single infection in a fully susceptible population without any disease control. In our model, $R_{\text{0}}$ is calculated as


$$\begin{array}{c} R_{\text{0}}=\frac{\beta \delta }{\delta +\mu }\left(\frac{\rho_{A}(\text{1}-k)}{\gamma_{A}+\mu }+\frac{k}{\gamma +\mu }\right). \end{array}$$ (4)

The effective reproduction number, $R_{e}(\phi )$, represents the average number of secondary cases generated by a single infection in the presence of vaccination, and is given by


$$\begin{array}{c} R_{e}=\frac{\beta \delta }{\delta +\mu }[(\text{1}-\phi )\left(\frac{(\text{1}-k)\rho_{A}}{\gamma_{A}+\mu }+\frac{k}{\gamma +\mu }\right)+\phi \left(\text{1}-\theta_{I}\right)\left(\text{1}-\theta_{T}\right)\left(\frac{\left\{\text{1}-\left(\text{1}-\theta_{S}\right)k\right\}\rho_{A}}{\gamma_{A}+\mu }+\frac{\left(\text{1}-\theta_{S}\right)k}{\gamma +\mu }\right)] \end{array}$$ (5)

Derivations of $R_{\text{0}}$ and $R_{e}$ are provided in the Appendix (S1 Appendix). When $R_{e}>\text{1}$, an endemic steady state exists, which can be derived by setting all differential equations in (1) and (2) to zero and solving for equilibrium values. The endemic steady state of our model is as follows: $\text{E}=\left(S_{U}^{*},\;E_{U}^{*},\;A_{U}^{*},\;I_{U}^{*},\;R_{U}^{*},\;S_{V}^{*},\;E_{V}^{*},\;A_{V}^{*},\;I_{V}^{*},\;R_{V}^{*}\right),$

where$\;S_{U}^{*}=\frac{\text{1}-\phi }{R_{e}},$


$$\begin{array}{c} E_{U}^{*}=\frac{\mu (\text{1}-\phi )}{\delta +\mu }\left(\text{1}-\frac{\text{1}}{R_{e}}\right), \end{array}$$

$$\begin{array}{c} A_{U}^{*}=\frac{\mu (\text{1}-\phi )\delta (\text{1}-k)}{(\delta +\mu )\left(\gamma_{A}+\mu \right)}\left(\text{1}-\frac{\text{1}}{R_{e}}\right), \end{array}$$

$$\begin{array}{c} I_{U}^{*}=\frac{\mu (\text{1}-\phi )\delta k}{\left(\delta +\mu )(\gamma +\mu \right)}\left(\text{1}-\frac{\text{1}}{R_{e}}\;\right), \end{array}$$

$$\begin{array}{c} R_{U}^{*}=\gamma_{A}\frac{\left(\text{1}-\phi )\delta (\text{1}-k\right)}{(\delta +\mu )\left(\gamma_{A}+\mu \right)}\left(\text{1}-\frac{\text{1}}{R_{e}}\right)+\gamma \frac{(\text{1}-\phi )\delta k}{\left(\delta +\mu )(\gamma +\mu \right)}\left(\text{1}-\frac{\text{1}}{R_{e}}\;\right)\;, \end{array}$$

$$\begin{array}{c} S_{V}^{*}=\frac{\phi (\text{1}-\theta_{I})}{R_{e}}, \end{array}$$

$$\begin{array}{c} E_{V}^{*}=\frac{\mu \phi \left(\text{1}-\theta_{I}\right)}{\delta +\mu }\left(\text{1}-\frac{\text{1}}{R_{e}}\right), \end{array}$$

$$\begin{array}{c} A_{V}^{*}=\frac{\mu \phi \left(\text{1}-\theta_{I}\right)\delta \left\{\text{1}-\left(\text{1}-\theta_{S}\right)k\right\}}{(\delta +\mu )\left(\gamma_{A}+\mu \right)}\left(\text{1}-\frac{\text{1}}{R_{e}}\right), \end{array}$$

$$\begin{array}{c} I_{V}^{*}=\frac{\mu \phi \left(\text{1}-\theta_{I}\right)\delta (\text{1}-\theta_{S})k}{\left(\delta +\mu )(\gamma +\mu \right)}\left(\text{1}-\frac{\text{1}}{R_{e}}\right), \end{array}$$

and $R_{V}^{*}=\gamma_{A}\frac{\phi \left(\text{1}-\theta_{I}\right)\delta \left\{\text{1}-\left(\text{1}-\theta_{S}\right)k\right\}}{(\delta +\mu )\left(\gamma_{A}+\mu \right)}\left(\text{1}-\frac{\text{1}}{R_{e}}\right)+\gamma \frac{\phi \left(\text{1}-\theta_{I}\right)\delta (\text{1}-\theta_{S})k}{\left(\delta +\mu )(\gamma +\mu \right)}\left(\text{1}-\frac{\text{1}}{R_{e}}\right)$

### Escape pressure

We define the escape pressure (P(t)) as the rate at which resistant pathogens emerge. We assume that escape pressure (P(t)) is linearly dependent on the number of infected cases at time t [8], represented as


$$\begin{array}{c} P(t)=\chi_{A}A_{U}(t)+I_{U}(t)+\xi \{\chi_{A}A_{V}(t)+I_{V}(t)\}\text{.} \end{array}$$ (6)

We note that P(t) represents a heuristic index quantifying the opportunity for immune escape, rather than predicting mutation or fixation probabilities or mechanistically simulating pathogen evolution. The parameter ξ indicates the relative contribution of vaccinated versus unvaccinated infections to escape pressure. Similarly, the parameter $\chi_{A}$ represents the relative contribution of asymptomatic infections to escape pressure compared to symptomatic infections.

Equation (6) shows that escape pressure is proportional to the number of infections, with ξ (vaccination status) and $\chi_{A}$ (symptom status) weighting contributions according to vaccination and symptom status, respectively. In equation (6) the effects of vaccination status (ξ) and symptom status ($\chi_{A}$) on escape pressure are assumed to be independent. All state variables are normalized by the total population size. Accordingly, when the weighting parameters (ξ and $\chi_{A}$) are set to 1, the escape pressure P(t) represents the proportion of infected cases in the population. In general, however, different infection types may contribute differently to immune escape due to differences in pathogen load and transmission potential. Bacterial pathogens such as *Bordetella pertussis* harbor genetic variation arising through point mutations, insertion sequence-mediated rearrangements, and gene loss events. Therefore, larger infected populations provide more opportunities for escape variant generation through these mutational processes [28,29].

Acellular pertussis vaccines target a limited set of antigens and may therefore impose selective pressure on specific vaccine-targeted epitopes. Breakthrough infections therefore create stronger within-host selection for escape variants compared to infections in immunologically naive hosts [30,31]. Genomic studies have reported accelerated accumulation of mutations in vaccine-antigen genes and progressive dominance of non-vaccine type strains in highly vaccinated populations [32–34]. We therefore set ξ=2 as our baseline and explore the sensitivity of our results to this assumption across a range of ξ values.

For $\chi_{A}$, we adopt the conservative baseline assumption that asymptomatic and symptomatic infections contribute equally to escape pressure ($\chi_{A}=\text{1}$). The instantaneous escape pressure P(t) reflects pathogen burden at a given time rather than cumulative shedding duration. Empirical evidence comparing within-host pathogen load between asymptomatic and symptomatic pertussis infections did not clearly support a higher instantaneous escape contribution from asymptomatic infections [20,22]. Accordingly, we set $\chi_{A}=\text{1}$ as our baseline and explore the sensitivity of our results to this parameter across a range of values. To explore how the escape pressure depends on the vaccination coverage (ϕ), we considered the asymptotic value of escape pressure P at the endemic equilibrium:


$$\begin{array}{c} P(\phi )=\underset{t\to \infty }{\text{lim}}P(t)=\chi_{A}A_{U}^{*}+I_{U}^{*}+\xi (\chi_{A}A_{V}^{*}+I_{V}^{*})\text{.} \end{array}$$ (7)

Therefore, if $R_{e}>\text{1}$, the escape pressure at endemic equilibrium is given by


$$\begin{array}{cc} P(\phi )=\; & \frac{\mu }{\delta +\mu }\left(\text{1}-\frac{\text{1}}{R_{e}}\right)[\frac{\chi_{A}}{\gamma_{A}+\mu }\left[(\text{1}-\phi )\delta (\text{1}-k)+\xi \phi \left(\text{1}-\theta_{I}\right)\delta \left\{\text{1}-\left(\text{1}-\theta_{S}\right)k\right\}\right]+\frac{\text{1}}{\gamma +\mu }\\ & \left\{(\text{1}-\phi )\delta k+\xi \phi \left(\text{1}-\theta_{I}\right)\delta \left(\text{1}-\theta_{S}\right)k\right\}] \end{array}$$

and P(ϕ)=0 for $R_{e}\leq \text{1}$.

To investigate how vaccination coverage level affects escape pressure P(ϕ), we defined the relative escape pressure $\hat{P}(\phi )$ as the ratio of the escape pressure with vaccination (ϕ>0) to that without vaccination (ϕ=0). This normalization gives


$$\begin{array}{cc} & \hat{P}(\phi )=\frac{P(\phi )}{P(\text{0})}=\\ & \;\;\;\left[\frac{\frac{\chi_{A}}{\gamma_{A}+\mu }\left[(\text{1}-\phi )\delta (\text{1}-k)+\xi \phi \left(\text{1}-\theta_{I}\right)\delta \left\{\text{1}-\left(\text{1}-\theta_{S}\right)k\right\}\right]+\frac{\text{1}}{\gamma +\mu }\left\{(\text{1}-\phi )\delta k+\xi \phi \left(\text{1}-\theta_{I}\right)\delta \left(\text{1}-\theta_{S}\right)k\right\}}{\frac{\chi_{A}}{\gamma_{A}+\mu }\left\{\delta (\text{1}-k)\right\}+\frac{\text{1}}{\gamma +\mu }(\delta k)}\right]\left(\frac{\text{1}-{R_{e}}^{-\text{1}}}{\text{1}-{R_{\text{0}}}^{-\text{1}}}\right) \end{array}$$ (8)

for $R_{e}>\text{1}$, and $\hat{P}(\phi )=\text{0}$ if $R_{e}\leq \text{1}$. Here, a value of $\hat{P}(\phi )>\text{1}$ implies that vaccination increases the escape pressure compared to no vaccination, while $\hat{P}(\phi )=\text{1}$ indicates that the escape pressure remains unchanged between the two scenarios.

## Results

Our analysis examined how vaccine efficacies ($\theta_{I}$, $\theta_{S}$, and $\theta_{T}$) and coverage (ϕ) affect relative escape pressure ($\hat{P}(\phi )$). As shown in Fig 2, the effects on escape pressure differed among the three efficacy parameters. Among the efficacy parameters, $\theta_{I}$ (protection against infection) exerted the strongest influence on escape pressure, directly preventing breakthrough infections and reducing the opportunities available for escape variant generation. This is evident in Fig 2A, where escape pressure falls steeply as $\theta_{I}$ increases. This effect is amplified by our assumption of ξ=2, under which breakthrough infections contribute twice as much to escape pressure as infections in unvaccinated individuals.

Vaccine efficacies against transmission ($\theta_{T}$) and symptoms ($\theta_{S}$) also reduced escape pressure by limiting pathogen spread and overall burden of infection, respectively. As shown in Fig 2C, the gradient along the $\theta_{T}$ axis is notably flat when vaccination coverage (ϕ) is low, indicating little change in escape pressure with increasing $\theta_{T}$. This is because transmission-blocking efficacy provides minimal benefit when applied to a small proportion of the population. The gradient along the $\theta_{S}$ axis is similarly weak at low coverage (Fig 2B), where escape pressure stays near its pre-vaccination level (yellow) across most of the $\theta_{S}$ range. Because asymptomatic infections clear more quickly and thus transmit for a shorter period, shifting infections from symptomatic to asymptomatic lowers the total number of infections at equilibrium, so $\theta_{S}$ reduces escape pressure even when symptomatic and asymptomatic infections contribute equally ($\chi_{A}=\text{1}$). The three efficacy components thus suppress escape pressure through distinct mechanisms. As seen in Fig 2, increasing vaccination coverage generally reduces escape pressure; a notable exception is found below the white dotted line in Fig 2B (low $\theta_{S}$), where escape pressure remains above the pre-vaccination level even at relatively high coverage.

In an additional analysis, we varied one efficacy parameter at a time while setting the other two to zero (S1 Fig). Escape pressure exceeded pre-vaccination levels primarily when multiple efficacy components were simultaneously low. The capacity of vaccination to suppress escape pressure depends on the overall magnitude of protection conferred across infection, symptom, and transmission pathways.

Fig 3 further illustrates the influence of the relative contribution of breakthrough infections (ξ) to escape pressure under varying coverage levels (ϕ), assuming the baseline value of symptom-blocking efficacy ($\theta_{S}=\text{0}.\text{8}\text{5}$), a relatively high level. Even when breakthrough infections contribute up to twice the escape pressure of infections in unvaccinated individuals (ξ≤2), any degree of vaccination coverage consistently reduces escape pressure below pre-vaccination levels, with $\hat{P}(\phi )=\text{1}$ only in the limiting case of no vaccination (ϕ=0).

Fig 4 examines the relationship between relative escape pressure in a fully vaccinated population, denoted as $\hat{P}(\text{1})$, and the vaccine efficacies $\theta_{S}$ and $\theta_{I}$. If full vaccination does not prevent an epidemic, i.e., $R_{e}(\phi =\text{1})>\text{1}$, then it follows that


$$\begin{array}{c} \hat{P}(\text{1})=\left[\frac{\frac{\chi_{A}}{\gamma_{A}+\mu }\left[\xi \left(\text{1}-\theta_{I}\right)\delta \left\{\text{1}-\left(\text{1}-\theta_{S}\right)k\right\}\right]+\frac{\text{1}}{\gamma +\mu }\left\{\xi \left(\text{1}-\theta_{I}\right)\delta \left(\text{1}-\theta_{S}\right)k\right\}}{\frac{\chi_{A}}{\gamma_{A}+\mu }\left\{\delta (\text{1}-k)\right\}+\frac{\text{1}}{\gamma +\mu }(\delta k)}\right]\left(\frac{\text{1}-{R_{e}}^{-\text{1}}}{\text{1}-{R_{\text{0}}}^{-\text{1}}}\right) \end{array}$$

In a full-vaccination scenario, escape pressure decreases with increasing $\theta_{I}$ and $\theta_{S}$, as both reducing breakthrough infections and suppressing symptomatic disease limit pathogen replication and thereby curtail opportunities for escape variant generation (Fig 4). However, even at full vaccination coverage, escape pressure can exceed pre-vaccination levels when both $\theta_{I}$ and $\theta_{S}$ are low — with the boundary defined by a trade-off between the two, such that higher $\theta_{S}$ can compensate for lower $\theta_{I}$ and vice versa. Furthermore, in the region where both $\theta_{I}$ and $\theta_{S}$ are simultaneously low, escape pressure in the fully vaccinated population can reach twice the pre-vaccination level (reflecting ξ=2).

**Fig 5:**
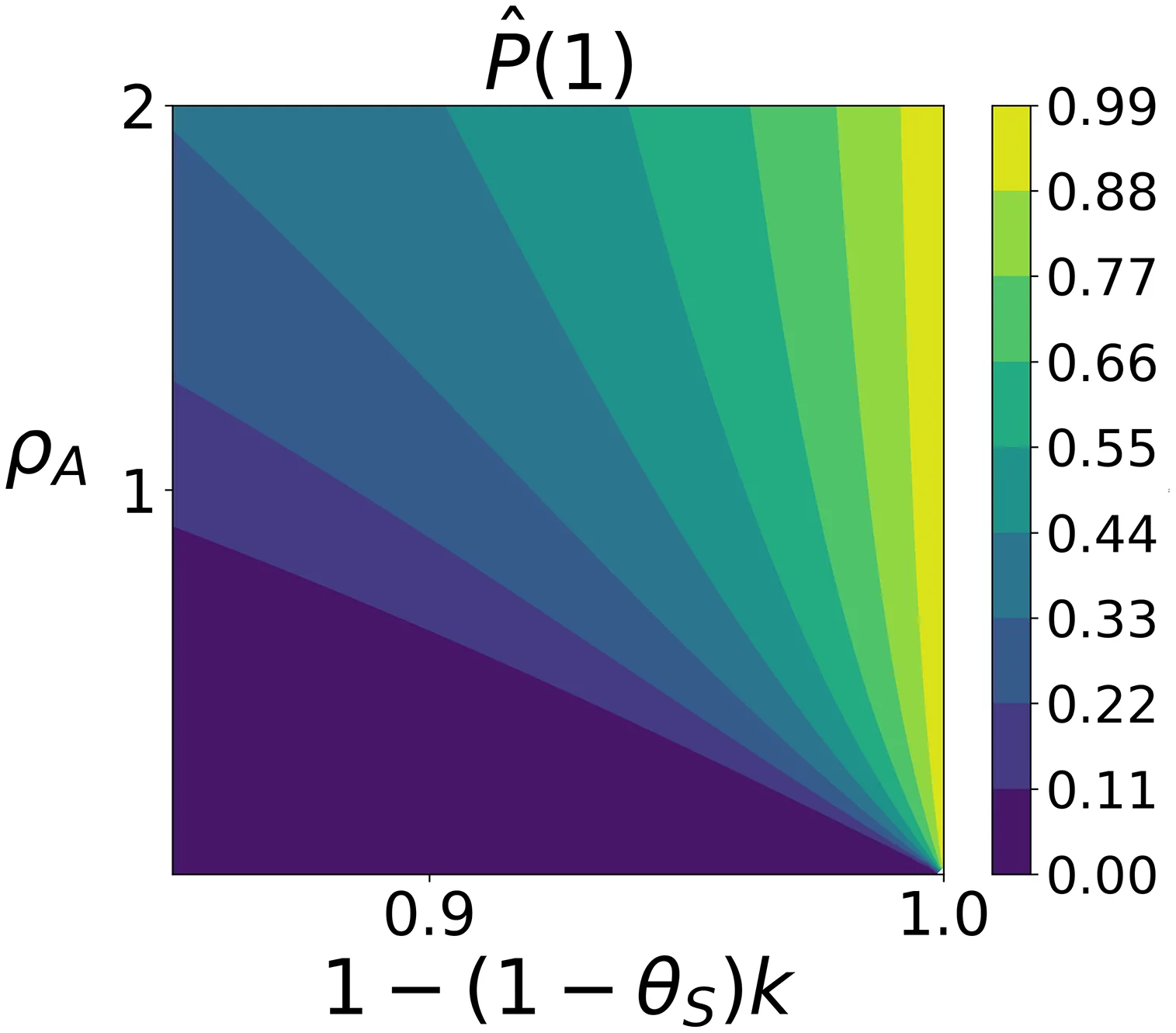
Relative escape pressure $\hat{P}\left(1\right)$ in a fully vaccinated population with varying asymptomatic infectiousness ($\rho_{A}$) and proportion of asymptomatic infections in the vaccinated population ($1-(1-\theta_{S})k$). Here, $\theta_{S}\;$is fixed at its baseline value, while k is varied from 0 to 1. Other parameters are set to the baseline values from Table 1.

Fig 5 examines how asymptomatic infections influence escape pressure in a fully vaccinated population through two mechanisms. First, we analyze the impact of the proportion of asymptomatic infections in the vaccinated infected population $\left(\text{1}-\left(\text{1}-\theta_{S}\right)k\right)$ — determined jointly by the proportion of exposed individuals who become symptomatic (k) and vaccine efficacy against symptoms ($\theta_{S}$) — on escape pressure. Second, we assess the impact of their relative infectiousness ($\rho_{A}$) compared to symptomatic infections on escape pressure at full vaccination coverage. Across the full parameter space explored, escape pressure in the fully vaccinated population remains below pre-vaccination levels, even when asymptomatic infections are up to twice as infectious as symptomatic infections ($\rho_{A}\leq \text{2}$) and regardless of their proportion among infections.

**Fig 6:**
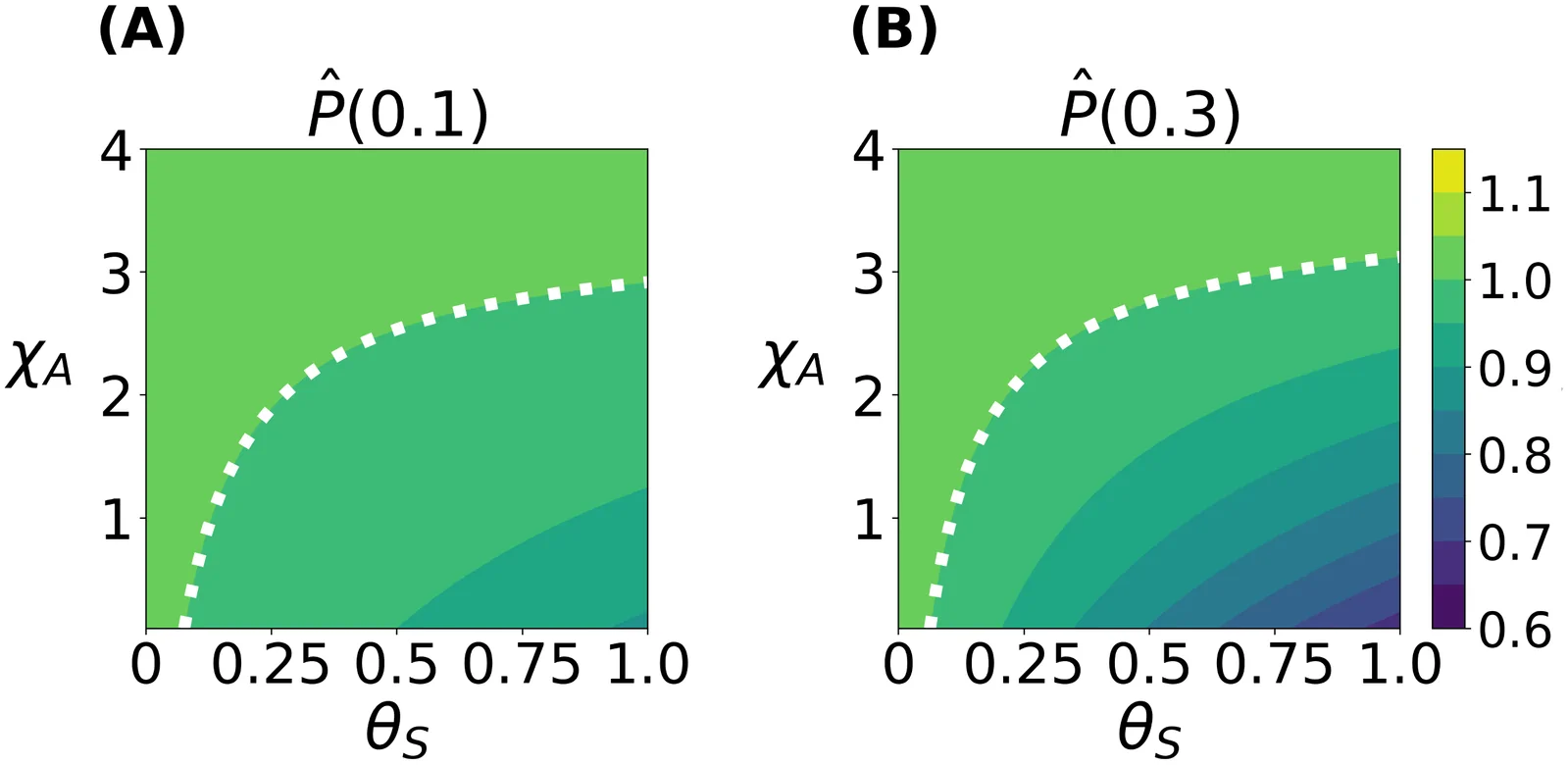
Relative escape pressure with varying vaccine efficacy and contribution of asymptomatic infections to escape pressure. (A) Relative escape pressure $\hat{P}(\text{0}.\text{1})$ with varying vaccine efficacy against symptoms ($\theta_{S}$) and relative contribution of asymptomatic infections to escape pressure ($\chi_{A}$) at a vaccine coverage level of 10%. (B) Relative escape pressure $\hat{P}(\text{0}.\text{3})$ with varying vaccine efficacy against symptoms ($\theta_{S}$) and relative contribution of asymptomatic infections to escape pressure ($\chi_{A}$) at a vaccine coverage level of 30%. The white dotted curve represents $\hat{P}(\phi )=\text{1}$, indicating that the escape pressure at vaccination coverage ϕ is the same as without vaccination. Other parameters are set to the baseline values from Table 1.

Fig 6 examines how the relative contribution of asymptomatic infections to escape pressure ($\chi_{A}$) interacts with vaccine efficacy against symptoms ($\theta_{S}$) at low (ϕ=0.1) and moderate (ϕ=0.3) coverage levels. A clear $\hat{P}(\phi )=\text{1}$ boundary emerges in both panels: below this boundary — at low $\chi_{A}$ and high $\theta_{S}$ — vaccination reduces escape pressure below pre-vaccination levels; above it — at high $\chi_{A}$ and low $\theta_{S}$ — escape pressure exceeds pre-vaccination levels. Escape pressure was highest when asymptomatic infections were disproportionately potent drivers of variant evolution and symptom-blocking immunity is limited. At moderate coverage (ϕ=0.3), the boundary shifts, expanding the region where vaccination effectively suppresses escape pressure, but the escape-amplifying zone at high $\chi_{A}$ persists. The effect of symptom-blocking efficacy on escape pressure therefore depended on the relative contribution of asymptomatic infections ($\chi_{A}$).

S1–S4 Figs show the effects of individual and paired efficacy components on escape pressure. When each component acts alone (S1 Fig), increasing coverage elevates escape pressure above pre-vaccination levels when efficacy was low. This counterintuitive result arises because a vaccine with insufficient efficacy fails to meaningfully reduce individual infection risk, yet expands the pool of breakthrough infections that contribute disproportionately to escape pressure (ξ=2). As a result, broader coverage under low efficacy conditions amplifies rather than suppresses escape risk. These results collectively highlight that expanding vaccination coverage without sufficient efficacy can be counterproductive in terms of escape pressure.

When efficacy components are combined in pairs at low coverage (ϕ=0.1), the parameter region with escape pressure above the pre-vaccination level was smaller. The combination of $\theta_{I}$ and $\theta_{T}$ (S2 Fig) suppresses escape pressure below pre-vaccination levels across the entire parameter space, indicating that even modest levels of each are jointly sufficient to eliminate escape risk. When $\theta_{I}$ and $\theta_{S}$ are combined (S3 Fig), or when $\theta_{T}$ and $\theta_{S}$ are combined (S4 Fig), escape pressure exceeds pre-vaccination levels only when both components are simultaneously low, with the $\hat{P}(\text{0}.\text{1})=\text{1}$ boundary tracing a compensatory trade-off — higher values of one component can offset lower values of the other. Taken together, these results demonstrate that the risk of vaccination increasing escape pressure is effectively mitigated when multiple efficacy components act in combination, underscoring that sufficient efficacy across multiple components is essential for ensuring that expanded coverage translates into reduced escape risk.

## Discussion

Our study reveals complex relationships between vaccine efficacies, coverage levels, and pathogen escape pressure. We emphasize that vaccination consistently reduces the absolute number of infections and disease burden, as it is straightforward to show that $\frac{d\left(I_{U}^{*}+I_{V}^{*}\right)}{d\phi }<\text{0}$. Our analysis is not intended to discourage vaccination, but rather to inform the design of vaccine profiles that minimize both disease burden and immune escape pressure simultaneously. While vaccines remain critical for controlling infectious diseases, their impact on pathogen evolution depends on specific efficacy dimensions: prevention of infection, reduction of symptoms, and limitation of transmission. Our findings demonstrate conditions where vaccination may either suppress or increase immune escape, with the latter scenario occurring when the vaccine efficacy profile is insufficient across multiple dimensions.

Our model is not intended to be universally applicable to all infectious diseases. In particular, it is not suitable for pathogens that are almost always symptomatic, such as measles, or for pathogens with mutation rates high enough to require annual vaccine updates, such as influenza. For these pathogens, immune escape is driven primarily by intrinsic mutation processes rather than by vaccine-induced escape pressure, which is beyond the scope of this study. Furthermore, pathogen-specific characteristics such as antigenic stability and the route of transmission may also affect pathogen evolutionary dynamics.

Among the three efficacy components, infection-blocking immunity had the largest effect on escape pressure. Even modest increases in infection-blocking efficacy substantially reduced escape pressure because each prevented infection removes a potential source of variant generation. This finding aligns with theoretical predictions from population genetics that fewer replication events reduce the probability that beneficial mutations arise and are selected [35,36]. Vaccine efficacy against transmission, $\theta_{T}$, similarly reduces escape pressure by curtailing onward pathogen spread. Combining infection- and transmission-blocking efficacy produced larger reductions than either component alone.

Vaccine efficacy against symptoms differs from the other two components in that its effectiveness depends on the overall vaccine efficacy profile, particularly infection-blocking efficacy. When asymptomatic and symptomatic infections contribute equally to escape pressure, efficacy against symptoms reduces escape pressure by diverting breakthrough infections toward asymptomatic pathways with shorter infectious duration (1/γA < 1/γ) — thereby reducing the time infections remain in the population and the cumulative pathogen burden — and its effect is further strengthened when combined with efficacy against infection or transmission. When asymptomatic infections contribute disproportionately to escape pressure ($\chi_{A}>\text{1}$), however, even high efficacy against symptoms may be insufficient to bring escape pressure below pre-vaccination levels, as symptom suppression cannot neutralize the escape contribution of a highly potent asymptomatic infectious pool. This finding underscores that the consequences of vaccine efficacy against symptoms for escape pressure cannot be assessed in isolation from the other efficacy components.

Even in a fully vaccinated population, escape pressure can exceed pre-vaccination levels — reaching up to twice the pre-vaccination level — when both efficacy against infection and efficacy against symptoms are simultaneously low. Although this result depends strongly on the assumption that breakthrough infections contribute disproportionately to escape pressure (ξ > 1), it highlights that full vaccination coverage does not inherently protect against elevated escape risk when the underlying efficacy profile is insufficient.

When vaccine efficacy is sufficient, expanding coverage monotonically reduces escape pressure. However, when efficacy is insufficient across multiple components simultaneously, increasing coverage can paradoxically elevate escape pressure above pre-vaccination levels, as broader coverage expands the pool of breakthrough infections that contribute disproportionately to escape pressure. Thus, the effect of increasing coverage depends on the underlying efficacy profile.

Our results extend and complement the work of Gutierrez and Gog [8] by adding compartments for asymptomatic infections, which we assume to be shorter than symptomatic infections. As discussed above, the reduced duration of asymptomatic infections reduces both infection load and escape pressure, unless asymptomatic infections contribute disproportionately to the latter ($\chi_{A}>\text{1}$). Our model extends their analysis by including asymptomatic infections and by varying the three vaccine efficacy components separately and in combination. This allowed us to examine how the contribution of asymptomatic infections modifies the effect of each efficacy component on escape pressure. Gutierrez and Gog also emphasize that escape pressure may be maximized at intermediate coverage levels, but this effect occurs only if the relative contribution of breakthrough infections to escape pressure (denoted ξ here and $\theta_{E}$ by Gutierrez and Gog) exceeds a relatively high threshold value (e.g., above 4 in their Fig 1b). In the present study, we focused on ξ between 0 and 2, for which escape pressure decreases with coverage for most parameter combinations.

Pertussis demonstrates how variants adapt despite widespread vaccination coverage, with several countries reporting disease resurgence [4]. Similarly, evidence from influenza research suggests that vaccination may accelerate short-term pathogen evolution when conferring partial immunity [37,38], and recent SARS-CoV-2 studies have documented higher mutation accumulation in vaccinated than in unvaccinated cases [39].

Our findings challenge the assumption that any level of vaccination will necessarily reduce variant evolution. When breakthrough infections exert significant selective pressure, vaccination alone may be insufficient to prevent immune escape in populations with moderate vaccine efficacy or incomplete coverage. This observation aligns with broader literature on vaccination showing that imperfect vaccines can potentially enhance transmission of highly virulent pathogens under certain conditions [6].

Despite these insights, our study has important limitations. First, we assumed vaccination occurs at birth, which may not reflect some real-world vaccination programs for respiratory pathogens such as influenza and SARS-CoV-2 that require repeated vaccination throughout life [40]. Age-stratified vaccination could significantly alter our conclusions, as different age groups exhibit varying immune responses, pathogen loads, and vaccine efficacy profiles. Our current model may overestimate escape pressure in populations with a large proportion of children, as children typically have lower pathogen loads and potentially generate fewer escape variants per infection. Conversely, in elderly populations with weaker vaccine responses, our model might underestimate escape pressure. Age-dependent variations in key parameters ($\chi_{A}$, ξ, $\theta_{I}$, $\theta_{S}$, and $\theta_{T}$) would require empirical characterization and could substantially modify our quantitative predictions about optimal vaccination strategies. Future studies should explore age-structured vaccination schedules and their implications for escape pressure. Second, our model assumes lifelong natural immunity after infection. Mathematical modeling of historical pertussis data suggests natural immunity lasts at least 30 years on average, potentially up to 70 years [41] — effectively approximating lifelong immunity. We used this assumption to isolate vaccine effects on escape pressure. However, we did not account for waning vaccine-induced immunity, which may significantly alter escape pressure as partially immune individuals could influence evolutionary dynamics [42]. Third, our linear definition of escape pressure, while following previous approaches [8], may not capture the full complexity of pathogen evolutionary dynamics. Alternative formulations could incorporate pathogen load, time-dependent weighting, or immunity-dependent scaling [43]. Fourth, our escape pressure metric is a heuristic proxy that is not derived from an explicit two-strain evolutionary model. Future models could explicitly include competing variants, fitness costs, and cross-immunity. Finally, although pertussis-specific parameters were used, our model is intended as a generalizable framework rather than a fully pertussis-tailored model. In particular, pertussis infection involves distinct phases — catarrhal, paroxysmal, and convalescent — with varying transmissibility across stages, which our current model does not capture. Incorporating phase-dependent transmissibility in future models would provide a more disease-tailored assessment of escape pressure.

These findings point to several priorities for future research and policy. Empirical characterization of vaccine efficacy profiles — specifically the three dimensions of infection-blocking, symptom-blocking, and transmission-blocking efficacy — is critical for predicting escape risk. Surveillance systems should be enhanced to monitor escape variant emergence, particularly focusing on contributions from symptomatic versus asymptomatic breakthrough infections. Our results suggest that vaccines with efficacy across multiple dimensions are more likely to reduce escape pressure than vaccines with strong efficacy in only one dimension.

In conclusion, the effects of vaccination on immune escape depend critically on the vaccine efficacy profile and coverage level. Ensuring that coverage expansion is accompanied by sufficient efficacy across infection-blocking, symptom-reducing, and transmission-limiting dimensions is essential for vaccination programs to minimize escape risk. Understanding these dynamics may help inform vaccine deployment and surveillance strategies for evolving pathogens.

## Supporting information

S1 Appendix Calculation of the reproduction number. (DOCX)

S1 FigImpact of vaccine efficacies and coverage level on relative escape pressure. Each figure shows the effect of varying only one vaccine efficacy parameter, while the other two were fixed at 0.The white dotted curve represents $\hat{P}(\phi )=1$, indicating that escape pressure at vaccination coverage level, ϕ, is equivalent to that without vaccination. (A) Relative escape pressure $\hat{P}(\phi )$ with varying vaccination coverage (ϕ) and vaccine efficacy to prevent infection ($\theta_{I}$) ($\theta_{S}=\theta_{T}=0$). (B) Relative escape pressure $\hat{P}(\phi )$ with varying vaccination coverage (ϕ) and vaccine efficacy to prevent symptomatic infection ($\theta_{S}$) ($\theta_{I}=\theta_{T}=0$). (C) Relative escape pressure $\hat{P}(\phi )$ with varying vaccination coverage (ϕ) and vaccine efficacy to reduce transmission of breakthrough infections ($\theta_{T}$) ($\theta_{S}=\theta_{I}=0$). Other parameters are set to the baseline values from Table 1.(PNG)

S2 FigRelative escape pressure $\hat{P}\left(0.1\right)$ with varying vaccine efficacy $\theta_{I}$ and $\theta_{T}$ compared to no vaccination.Other parameters are set to baseline values that are referenced from Table 1.(PNG)

S3 FigRelative escape pressure $\hat{P}\left(0.1\right)$ with varying vaccine efficacy $\theta_{I}$ and $\theta_{S}$ compared to no vaccination.The white dotted curve represents $\hat{P}(0.1)=1$, indicating that the escape pressure at vaccination coverage ϕ=0.1 is the same as without vaccination. Other parameters are set to baseline values that are referenced from Table 1.(PNG)

S4 FigRelative escape pressure $\hat{P}\left(0.1\right)$ with varying vaccine efficacy $\theta_{T}$ and $\theta_{S}$ compared to no vaccination.The white dotted curve represents $\hat{P}(0.1)=1$, indicating that the escape pressure at vaccination coverage ϕ=0.1 is the same as without vaccination. Other parameters are set to baseline values that are referenced from Table 1.(PNG)

S5 Fig Bifurcation of symptomatic infections in the unvaccinated population according to vaccination coverage. The blue line represents the disease-free equilibrium, and the red line represents the endemic equilibrium. The solid line indicates that the equilibrium is stable, whereas the dashed line indicates that the equilibrium is unstable. The disease-free equilibrium remains unstable throughout the vaccination coverage, while the endemic equilibrium remains stable.
(PNG)

S6 Fig Bifurcation of symptomatic infections in the vaccinated population according to vaccination coverage. The blue line represents the disease-free equilibrium, and the red line represents the endemic equilibrium. The solid line indicates that the equilibrium is stable, whereas the dashed line indicates that the equilibrium is unstable. The disease-free equilibrium remains unstable throughout the vaccination coverage, while the endemic equilibrium remains stable.
(PNG)
