# Supplementary material for: Breakthrough infections and incomplete vaccine efficacy drive pathogen immune escape
Source: PLoS One. 2026 Sep 2;21(9):e0356544. doi: 10.1371/journal.pone.0356544 (PMC13537599; doi:10.1371/journal.pone.0356544)
Supplement: Supplementary file 1 [file pone.0356544.s001.docx]

**S1 Appendix. Calculation of the basic reproduction number**

**Calculation of the basic reproduction number**

The basic reproduction number, $R_{0}$, is defined as the average number of secondary cases generated by a single infected individual without any disease control. To derive $R_{0}$, we consider the following system of ordinary differential equations (ODEs) governing the dynamics of susceptible, exposed, infectious, and recovered individuals in the unvaccinated population:

For unvaccinated individuals:

$\dot{S}_{U}=\mu-\left( \lambda\left( t \right)+\mu\right)S_{U}$

$\dot{E}_{U}=\lambda\left( t \right)S_{U}-\left( \delta+\mu\right)E_{U}$

$\dot{A}_{U}=\delta\left( 1-k \right)E_{U}-\left( \gamma_{A}+\mu\right)A_{U}$

$\dot{I}_{U}=\delta kE_{U}-\left( \gamma+\mu\right)I_{U}$

$\dot{R}_{U}=\gamma_{A}A_{U}+\gamma I_{U}-\mu R_{U}$	(1)

where the force of infection is $\lambda(t)=\beta(\rho_{A}A_{U}+I_{U})$.

We apply the next-generation approach to compute $R_{0}$ [1]. First, we divide the system into infected compartments ($E_{U}$, $A_{U}$, $I_{U}$) and noninfected compartments ($E_{V}$, $A_{V}$, $I_{V}$) and write the system in the form $x_{i}^{'}=f_{i}(x,y)$ and $y_{j}^{'}=g_{j}\left( x,y \right)$:

$x_{1}^{'}=\dot{E}_{U}=\lambda\left( t \right)S_{U}-\left( \delta+\mu\right)E_{U}=\beta(\rho_{A}A_{U}+I_{U})S_{U}-\left( \delta+\mu\right)E_{U}$

$x_{2}^{'}=\dot{A}_{U}=\delta\left( 1-k \right)E_{U}-\left( \gamma_{A}+\mu\right)A_{U}$

$x_{3}^{'}=\dot{I}_{U}=\delta kE_{U}-\left( \gamma+\mu\right)I_{U}$

$y_{1}^{'}=\dot{S}_{U}=\mu-\left( \lambda\left( t \right)+\mu\right)S_{U}$

$y_{2}^{'}=\dot{R}_{U}=\gamma_{A}A_{U}+\gamma I_{U}-\mu R_{U}$

Next, we split each infected equation into two components of $\mathcal{F}_{i}(x,y)$ which is the rate of appearance of new infections, and $\mathcal{V}_{i}(x,y)$ which incorporates the remaining transitional terms.

$x_{1}^{'}=\dot{E}_{U}=\mathcal{F}_{1}\left( x,y \right)-\mathcal{V}_{1}\left( x,y \right)=\beta(\rho_{A}A_{U}+I_{U})S_{U}-\left( \delta+\mu\right)E_{U}$

$x_{2}^{'}=\dot{A}_{U}=\mathcal{F}_{2}\left( x,y \right)-\mathcal{V}_{2}\left( x,y \right)=\delta\left( 1-k \right)E_{U}-\left( \gamma_{A}+\mu\right)A_{U}$

$x_{3}^{'}=\dot{I}_{U}=\mathcal{F}_{3}\left( x,y \right)-\mathcal{V}_{3}\left( x,y \right)=\delta kE_{U}-\left( \gamma+\mu\right)I_{U}$

where

$\mathcal{F}_{1}\left( x,y \right)=\beta(\rho_{A}A_{U}+I_{U})S_{U}$

$\mathcal{V}_{1}\left( x,y \right)=\left( \delta+\mu\right)E_{U}$

$\mathcal{F}_{2}\left( x,y \right)=0$

$\mathcal{V}_{2}\left( x,y \right)=-\delta\left( 1-k \right)E_{U}+\left( \gamma_{A}+\mu\right)A_{U}$

$\mathcal{F}_{3}\left( x,y \right)=0$

$\mathcal{V}_{3}\left( x,y \right)=-\delta kE_{U}+\left( \gamma+\mu\right)I_{U}$

At the disease-free equilibrium $\epsilon_{0}=\left( 0,y_{0} \right)=(0,0,0,1,0)$, the matrices $F=\left[ \frac{\partial\mathcal{F}_{i}\left( 0,y_{0} \right)}{\partial x_{j}} \right]$ and $V=\left[ \frac{\partial\mathcal{V}_{i}\left( 0,y_{0} \right)}{\partial x_{j}} \right]$ are $F=\left[ \begin{matrix} 0 & \beta\rho_{A} & \beta\\ 0 & 0 & 0 \\ 0 & 0 & 0 \end{matrix} \right]$ and $V=\left[ \begin{matrix} \delta+\mu& 0 & 0 \\ -\delta\left( 1-k \right) & \gamma_{A}+\mu& 0 \\ -\delta k & 0 & \gamma+\mu\end{matrix} \right]$.

Finally, the next-generation matrix $K$ is $K=FV^{-1}=\left[ \begin{matrix} 0 & \beta\rho_{A} & \beta\\ 0 & 0 & 0 \\ 0 & 0 & 0 \end{matrix} \right]\left[ \begin{matrix} \frac{1}{\delta+\mu} & 0 & 0 \\ \frac{\delta\left( 1-k \right)}{(\delta+\mu)(\gamma_{A}+\mu)} & \frac{1}{\gamma_{A}+\mu} & 0 \\ \frac{\delta k}{(\delta+\mu)(\gamma+\mu)} & 0 & \frac{1}{\gamma+\mu} \end{matrix} \right]$ and the spectral radius of $K$ is $\frac{\beta\delta}{\delta+\mu}\left( \frac{\rho_{A}\left( 1-k \right)}{\gamma_{A}+\mu}+\frac{k}{\gamma+\mu} \right)$. Therefore, the basic reproduction number $R_{0}=\frac{\beta\delta}{\delta+\mu}\left( \frac{\rho_{A}\left( 1-k \right)}{\gamma_{A}+\mu}+\frac{k}{\gamma+\mu} \right)$.

**Calculation of the effective reproduction number**

The effective reproduction number, $R_{e}$, is defined as the average number of secondary cases generated by a single infected individual in the presence of any disease control. We extended the system of ODEs to include vaccinated individuals:

For unvaccinated individuals:

$\dot{S}_{U}=\mu(1-\phi)-\left( \lambda\left( t \right)+\mu\right)S_{U}$

$\dot{E}_{U}=\lambda\left( t \right)S_{U}-\left( \delta+\mu\right)E_{U}$

$\dot{A}_{U}=\delta\left( 1-k \right)E_{U}-\left( \gamma_{A}+\mu\right)A_{U}$

$\dot{I}_{U}=\delta kE_{U}-\left( \gamma+\mu\right)I_{U}$

$\dot{R}_{U}=\gamma_{A}A_{U}+\gamma I_{U}-\mu R_{U}$	(1)

For vaccinated individuals:

$\dot{S}_{V}=\mu\phi(1-\theta_{I})-\left( \lambda\left( t \right)+\mu\right)S_{V}$

$\dot{E}_{V}=\lambda\left( t \right)S_{V}-\left( \delta+\mu\right)E_{V}$

$\dot{A}_{V}=\delta\left\{ 1-\left( 1-\theta_{S} \right)k \right\}E_{V}-\left( \gamma_{A}+\mu\right)A_{V}$

$\dot{I}_{V}=\delta\left( 1-\theta_{S} \right)kE_{V}-\left( \gamma+\mu\right)I_{V}$

$\dot{R}_{V}=\gamma_{A}A_{V}+\gamma I_{V}-\mu R_{V}$	(2)

The force of infection is $\lambda(t)=\beta\{\rho_{A}A_{U}+I_{U}+(1-\theta_{T})(\rho_{A}A_{V}+I_{V})\}$.

We used the same method described above. In this case, we write the original ODE system as follows:

$x_{1}^{'}=\dot{E}_{U}=\lambda\left( t \right)S_{U}-\left( \delta+\mu\right)E_{U}$

$x_{2}^{'}=\dot{A}_{U}=\delta\left( 1-k \right)E_{U}-\left( \gamma_{A}+\mu\right)A_{U}$

$x_{3}^{'}=\dot{I}_{U}=\delta kE_{U}-\left( \gamma+\mu\right)I_{U}$

$x_{4}^{'}=\dot{E}_{V}=\lambda\left( t \right)S_{V}-\left( \delta+\mu\right)E_{V}$

$x_{5}^{'}=\dot{A}_{V}=\delta\left\{ 1-\left( 1-\theta_{S} \right)k \right\}E_{V}-\left( \gamma_{A}+\mu\right)A_{V}$

$x_{6}^{'}=\dot{I}_{V}=\delta\left( 1-\theta_{S} \right)kE_{V}-\left( \gamma+\mu\right)I_{V}$

$y_{1}^{'}=\dot{S}_{U}=\mu(1-\phi)-\left( \lambda\left( t \right)+\mu\right)S_{U}$

$y_{2}^{'}={\dot{R}_{U}=\gamma}_{A}A_{U}+\gamma I_{U}-\mu R_{U}$

$y_{3}^{'}=\dot{S}_{V}=\mu\phi(1-\theta_{I})-\left( \lambda\left( t \right)+\mu\right)S_{V}$

$y_{4}^{'}=\dot{R}_{V}=\gamma_{A}A_{V}+\gamma I_{V}-\mu R_{V}$

Following the same next-generation matrix as above, the disease-free equilibrium is $\epsilon_{0}=\left( 0,y_{0} \right)=(0,0,0,0,0,0,1-\phi,0,\phi\left( 1-\theta_{I} \right),0)$.

The matrix of new infections and other transitions are given by

$F=\left[ \begin{matrix} 0 & \beta\rho_{A}(1-\phi) & \beta(1-\phi) & 0 & \beta\left( 1-\theta_{T} \right)\rho_{A}(1-\phi) & \beta(1-\theta_{T})(1-\phi) \\ 0 & 0 & 0 & 0 & 0 & 0 \\ 0 & 0 & 0 & 0 & 0 & 0 \\ 0 & \beta\rho_{A}\phi(1-\theta_{I}) & \beta\phi(1-\theta_{I}) & 0 & \beta\left( 1-\theta_{T} \right)\rho_{A}\phi(1-\theta_{I}) & \beta\left( 1-\theta_{T} \right)\phi(1-\theta_{I}) \\ 0 & 0 & 0 & 0 & 0 & 0 \\ 0 & 0 & 0 & 0 & 0 & 0 \end{matrix} \right]$ and $V=\left[ \begin{matrix} \delta+\mu& 0 & 0 & 0 & 0 & 0 \\ -\delta(1-k) & \gamma_{A}+\mu& 0 & 0 & 0 & 0 \\ -\delta k & 0 & \gamma+\mu& 0 & 0 & 0 \\ 0 & 0 & 0 & \delta+\mu& 0 & 0 \\ 0 & 0 & 0 & -\delta\{1-\left( 1-\theta_{S} \right)k\} & \gamma_{A}+\mu& 0 \\ 0 & 0 & 0 & -\delta\left( 1-\theta_{S} \right)k & 0 & \gamma+\mu\end{matrix} \right]$.

The next-generation matrix is derived as $K=FV^{-1}$. The spectral radius of $K$ is $\frac{\beta\delta}{\delta+\mu}[\left( 1-\phi\right)\left( \frac{\left( 1-k \right)\rho_{A}}{\gamma_{A}+\mu}+\frac{k}{\gamma+\mu} \right)+\phi\left( 1-\theta_{I} \right)\left( 1-\theta_{T} \right)\left( \frac{\left\{ 1-\left( 1-\theta_{S} \right)k \right\}\rho_{A}}{\gamma_{A}+\mu}+\frac{\left( 1-\theta_{S} \right)k}{\gamma+\mu} \right)$. Therefore, the effective reproduction number $R_{e}=\frac{\beta\delta}{\delta+\mu}[\left( 1-\phi\right)\left( \frac{\left( 1-k \right)\rho_{A}}{\gamma_{A}+\mu}+\frac{k}{\gamma+\mu} \right)+\phi\left( 1-\theta_{I} \right)\left( 1-\theta_{T} \right)\left( \frac{\left\{ 1-\left( 1-\theta_{S} \right)k \right\}\rho_{A}}{\gamma_{A}+\mu}+\frac{\left( 1-\theta_{S} \right)k}{\gamma+\mu} \right)$.

**References**

1. van den Driessche P, Watmough J. Reproduction numbers and sub-threshold endemic equilibria for compartmental models of disease transmission. Math Biosci. 2002;180:29-48.
